# Optimal Sleep Duration in the Subarctic with Respect to Obesity Risk Is 8–9 Hours

**DOI:** 10.1371/journal.pone.0056756

**Published:** 2013-02-15

**Authors:** May Trude Johnsen, Rolf Wynn, Trond Bratlid

**Affiliations:** 1 Department of Clinical Medicine, Faculty of Health Sciences, University of Tromsø, Tromsø, Norway; 2 University Hospital of North Norway, Tromsø, Norway; University College London, United Kingdom

## Abstract

**Introduction:**

Sleep duration, chronotype and social jetlag have been associated with body mass index (BMI) and abdominal obesity. The optimal sleep duration regarding BMI has previously been found to be 7–8 hours, but these studies have not been carried out in the subarctic or have lacked some central variables. The aims of our study were to examine the associations between sleep variables and body composition for people living in the subarctic, taking a range of variables into consideration, including lifestyle variables, health variables and biological factors.

**Methods:**

The cross sectional population Tromsø Study was conducted in northern Norway, above the Arctic Circle. 6413 persons aged 30–65 years completed questionnaires including self-reported sleep times, lifestyle and health. They also measured height, weight, waist and hip circumference, and biological factors (non-fasting serum level of cholesterol, HDL-cholesterol, LDL-cholesterol, triglycerides and glucose). The study period was from 1 October 2007 to 19 December 2008.

**Results:**

The optimal sleep length regarding BMI and waist circumference was found to be 8–9 hours. Short sleepers (<6 h) had about 80% increased risk of being in the BMI≥25 kg/m2 group and male short sleepers had doubled risk of having waist circumference ≥102 cm compared to 8–9 hours sleepers. We found no impact of chronotype or social jetlag on BMI or abdominal obesity after controlling for health, lifestyle, and biological parameters.

**Conclusions:**

In our subarctic population, the optimal sleep duration time regarding risk of overweight and abdominal obesity was 8–9 hours, which is one hour longer compared to findings from other studies. Short sleepers had 80% increased risk of being overweight, and men had a doubled risk of having abdominal obesity. We found no associations between chronotype or social jetlag and BMI or abdominal obesity, when we took a range of life-style, health and biological variables into consideration.

## Introduction

Many studies have indicated associations between short sleep duration and different health outcomes like total mortality, type 2 diabetes mellitus, cardiovascular disease and self-rated health [1]–[5]. Associations between short sleep duration and increased risk for weight gain and obesity have also been found [6]–[11], but the causal direction has not been evident.

Body mass index (BMI) has most commonly been used to measure obesity, but both skin fold thickness and waist and hip circumference can provide useful information [6], [12], [13]. Some reviews report no association between short sleep duration and increased BMI or waist circumference [14], or inconclusive results in adults [15]–[17]. Not only sleep duration, but also inadequate sleep timing and the role of social jetlag have been associated with increased vulnerability to obesity [18]. Social jetlag is referred to as the mismatch between the body's internal clock and social schedules, and can lead to chronic loss of sleep [19], [20].

Gender differences in the association between sleep duration and body composition have also been found [21], including positive associations between BMI and short sleep in women [22], [23] and associations between shorter sleep duration and higher BMI, waist circumference and subcutaneous fat area in men [24]. Increased abdominal fat has been related to a higher risk of death from major specific causes, independent of BMI [13].

Sleep timing and sleep duration are determined by biological and social factors such as daylight, temperature, social schedules, nutrition and health conditions [25], [26], and social schedules interfere with sleep preferences in the population [27]. In our recent study [28], we found that other factors may be more important than daylight exposure in the regulation of sleep patterns for the subarctic people.

The interactions of multiple biological, psychological, behavioral and social risk factors may play an important role in the development of overweight and obesity [29]–[32]. Our study is one of very few studies that have included sleep patterns, lifestyle factors, physical and psychological health factors and biological factors in relation to body composition variables.

In this article, we analysed the association between different body composition measures and sleep variables in the 6^th^ part of The Tromsø Study. We hypothesize that the associations between sleep variables and body composition parameters become weaker or disappear when health variables, lifestyle variables, and biological parameters are controlled for.

## Materials and Methods

### Ethics statement

The study was approved by the Regional Medical Ethics Committee North (REC North, based in Tromsø).

The participants provided their written informed consent to perform the procedures in this study, and retained the right to withdraw this consent at a later stage. This procedure is approved by the Regional Medical Ethics Committee North (REC North, based in Tromsø).

### Study population

The Tromsø Study is a population-based prospective study which was initiated in 1974 to investigate the high mortality due to cardiovascular diseases in Norway [33]. Our study was part of the 6^th^ Tromsø Study, which included entire birth cohorts and random samples of the population of Tromsø, aged 30 years or more. The data collection period was from 1 October 2007 to 19 December 2008, and a total of 12984 subjects participated (65.7% of the invited population). The participants completed two questionnaires, including questions about socioeconomic status, general health and diseases, sleep variables, mental distress, use of medication, smoking and alcohol habits, and physical activity in leisure and work.

### Measurements

#### Sleep variables

To examine sleep duration and timing, a Norwegian translation of The Munich ChronoType Questionnaire (MCTQ) was used [34]. The 15-items questionnaire included questions about shift work, the number of work days and free time during the week, and bedtime and rise time including sleep latency (in minutes) during work days and days off. Mean sleep duration was calculated as sleep duration on work days×number of work days plus sleep duration on free days×number of free days/7. We grouped mean sleep duration into 5 categories: <6 hours, 6–6.9 hours, 7–7.9 hours, 8–8.9 hours and ≥9 hours. Chronotype refers to the timing of sleep, i.e. if we become active early or late in the day. The quantification of chronotype is based on calculation of mid sleep, which is the half-way point between sleep onset and wake up time (local time). Most people accumulate a sleep loss on work days, which is compensated for on days off, and therefore we adjusted the midpoint of sleep on free days (MSFsc) for individual sleep needs as follows: MSFsc = mid sleep on free days – 0.5 (sleep duration on free days – mean sleep duration) [35]. Social jetlag was calculated as the difference between mid-sleep on free days and mid-sleep on work days (MSFsc-MSW) for those who worked. Negative values on social jetlag refer to participants who had earlier mid sleep on free days than on work days.

#### Body composition variables

Specially trained personnel measured height and weight with participants wearing light clothing and no shoes. Body mass index (BMI) was calculated as weight in kilograms (kg) divided by the square of the height in meters. Normal weight was defined as BMI<25 kg/m2 and overweight as BMI≥25 kg/m2. Waist circumference in centimeter (cm) was measured at the umbilical line and hip circumference in cm at the widest point. Waist-to-Hip Ratio (WHR) was calculated as waist circumference divided by hip circumference. Abdominal obesity was defined as waist circumference ≥102 cm for men and ≥88 cm for women.

#### HSCL-10

We used the Hopkins Symptoms Checklist for detecting symptoms of mental distress during the last week before admission [36]. The mean HSCL-10 score was calculated dividing the total score by ten (the number of items). Subjects with missing values have been excluded in the mean item score calculation. A mean score of 1.85 or higher indicated high mental distress.

#### Biological factors

Non-fasting serum level of total cholesterol, HDL-cholesterol, LDL-cholesterol, triglycerides and glucose were analysed.

A total of 8978 subjects completed the MCTQ. We excluded persons older than 65 years (n = 1686), participants who reported shift work (816), pregnant women (n = 18), subjects referring sleep duration on work days <3 hours (n = 18) or >13 hours (n = 0), subjects referring sleep duration on free days <3 hours (n = 16), subjects referring mean sleep duration <3 hours (n = 0) or >13 hours (n = 2), MSF>12:00 (n = 2) and social jetlag <3 hours (n = 8). Analyses were performed on a total of 6412 subjects 30 to 65 years of age.

### Statistical analyses

Univariate analyses were performed using One way ANOVAs for normally distributed variables and Kruskal-Wallis tests for variables that were not normally distributed. In the first univariate analyses between the different body composition variables and the sleep duration categories, we used 7–7.9 hours of sleep as the reference category. Age correction in univariate analyses was done by calculating the variable mean, by age. In regression analyses, we used ‘Age’ as a prediction variable in the model. Stepwise backwards multivariable regression analyses were used for adjustments. BMI, BMI≥25 kg/m2, abdominal obesity (measured by waist circumference) and WHR were set as outcome variables. Potential confounding factors in the regression analyses were: (i) Socioeconomic variables (age, sex, educational level, income level, living with spouse), (ii) Lifestyle variables (current smoking, alcohol habits, physical activity in leisure and work), (iii) Health variables (self –rated physical health, former/current heart disease/diabetes, HSCL-10 score, coping, use of painkillers, antidepressants and sleeping pills, measured blood pressure, (iv) Biological factors (non-fasting serum level of cholesterol, HDL-cholesterol, LDL-cholesterol, triglycerides and glucose) and (v) Sleep variables (mean sleep duration, chronotype and social jetlag). The statistical significance level was set at 0.05.

Analyses were performed with STATA version 11.1 (StataCorp LP, 4905 Lakeway Drive College Station, Texas, USA).

## Results

We found that men had a higher age-corrected BMI than women. 70% of men and 55% of women had a BMI≥25 kg/m2. More men were also classified as being obese (BMI≥30 kg/m2), but abdominal obesity was more common among women. The body composition variables are shown in Table 1.

**Table 1:** Body composition.

	All	Men	Women	Gender difference^1^
**BMI (kg/m2)** 2	26.7	27.3	26.3	**
**BMI>25 kg/m2 (%)** 3	62.6	71.8	54.3	**
**BMI≥30 kg/m2 (%)** 3	18.7	19.6	17.9	**
**Waist circumference (cm)** 1	94.2	98.8	90.0	**
**Abdominal obesity (%)** 3 ^,^ 4	46.2	37.4	54.1	**
**Waist-Hip-Ratio** 2	0.91	0.95	0.87	**

The table shows age corrected body composition variables between men and women.

1 Kruskal-Wallis test: ** = p<0.001, * = p<0.05.

2 Age corrected mean.

3 Age corrected rate (%).

4 Abdominal obesity: Male waist circumference ≥102 cm. Female waist circumference ≥88 cm.

Mean sleep duration was 7.04 hours (95% CI = 7.01–7.08 hours) for men and 7.25 hours (95% CI = 7.22–7.28 hours) for women. Univariate analyses (by Kruskal-Wallis tests) showed significant associations between body composition variables (BMI, BMI≥25 kg/m2 and waist circumference) and sleep duration, chronotype and social jetlag, as illustrated in Table 2.

**Table 2:** Body composition and sleep variables.

	Sleep duration^1^		Chronotype^2^		Social jetlag	
	*χ2* 1	*p*	*χ2* 1	*P*	*χ2* 1	*p*
***MALE***						
***BMI*** 2	*432.776*	*<0.001*	*252.069*	*<0.001*	*28.754*	*<0.001*
***BMI≥25 kg/m2*** 3	*205.160*	*<0.001*	*135.408*	*<0.001*	*29.406*	*<0.001*
***WHR*** 4	*288.595*	*<0.001*	*124.148*	*<0.001*	*171.180*	*<0.001*
***Abdominal obesity*** 5	*295.833*	*<0.001*	*28.756*	*<0.001*	*57.339*	*<0.001*
***FEMALE***						
***BMI*** 2	*265.889*	*<0.001*	*11.469*	*0.003*	*34.695*	*<0.001*
***BMI≥25 kg/m2*** 3	*197.065*	*<0.001*	*9.043*	*0.011*	*37.766*	*<0.001*
***WHR*** 5	*131.093*	*<0.001*	*Ns*		*16.667*	*<0.001*
***Abdominal obesity^6^***	*178.878*	*<0.001*	*40.700*	*<0.001*	*Ns*	

Univariate analyses between BMI and sleep variables, calculated by Kruskal-Wallis tests.

1 Kruskal-Wallis test.

2 Age corrected BMI.

3 Age corrected rate.

4 Age corrected Waist-Hip-Ratio.

5 Age corrected abdominal obesity: male waist ≥102 cm, female waist ≥88 cm.

**Figure 1:**
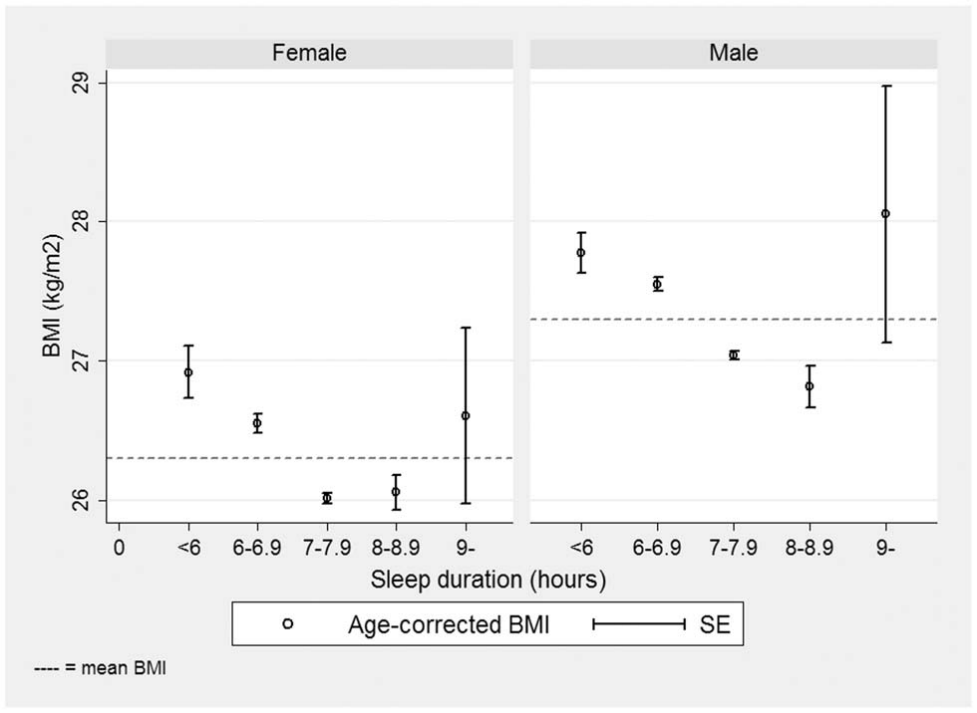
BMI by gender and sleep duration. The figure shows gender differences in age-corrected BMI by sleep duration groups (<6, 6–6.9, 7–7.9, 8–8.9 and ≥9 h).

Univariate analyses showed that BMI was increased for short sleepers (<6 hours) compared to 7–7.9 hours sleepers and 8–8.9 hours sleepers (see Table 3 and Figure 1).

**Table 3:** Body composition and sleep duration categories.

Sleep duration	<6	6–6.9	7–7.9	8–8.9	≥9
**MALE N**	305	1084	1339	287	38
**BMI** 1	27.78 (1.26)**	27.55 (0.84)**	27.04 (0.58) *	26.82 (1.29)† 3	28.05 (2.81)**
**BMI≥25 kg/m2** 2	0.744 (0.124)**	0.746 (0.077)**	0.701 (0.081) **	0.662 (0.149)† 3	0.684 (0.309)
**Waist-Hip-Ratio** 1	0.959 (0.023)**	0.948 (0.018)**	0.940 (0.018)†	0.945 (0.027) *	0.960 (0.042)**
**Abdominal obesity** 2	0.46 (0.169)**	0.389 (0.113)**	0.339 (0.107)†	0.364 (0.157)	0.553 (0.376)**
**FEMALE N**	242	896	1650	479	89
**BMI** 1	26.92 (1.459)**	26.55 (1.050)**	26.02 (0.825)	26.06 (1.412)† 3	26.61 (2.999)*
**BMI≥25 kg/m2** 2	0.603 (0.190)**^/^	0.575 (0.121)**	0.525 (0.096)	0.511 (0.157)† 3	0.562 (0.326)*
**Waist-Hip-Ratio** 1	0.877 (0.024)**	0.868 (0.016)*	0.866 (0.014)†	0.872 (0.020) **	0.877 (0.034)**
**Abdominal obesity** 2	0.595 (0.188)**	0.554 (0.122)**	0.518 (0.110)†	0.566 (0.161) **	0.545 (0.327)

The table shows associations between the different sleep durations, calculated by Kruskal-Wallis tests.

† Reference group.

1 Age corrected mean (SD).

2 Age corrected rate (SD).

3 Significant differences between the <6 h sleep group and the 8–8.9 h sleep group.

Statistical significance is marked * for p<0.05, and ** for p<0.001.

**Figure 2:**
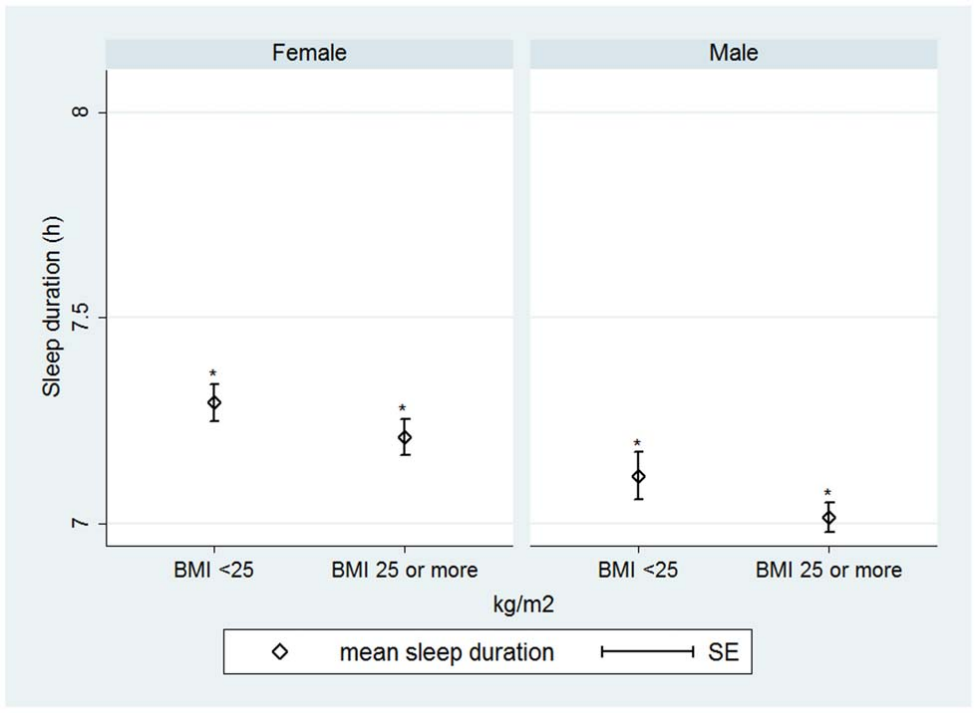
Sleep duration and overweight risk. The figure shows mean sleep duration between persons with normal weight (BMI<25 kg/m2) and overweight (BMI≥25 kg/m2). Statistically significant differences are marked with * (p<0.05).

We also examined the difference in mean sleep duration between the normal weight group and the group with BMI≥25 kg/m2 (see Figure 2). We found significant differences in mean sleep duration between the two groups, both for men (Kruskal-Wallis test: χ^2^ = 12.44, p<0.001) and women (Kruskal-Wallis test: χ^2^ = 9.02, p<0.05).

Next, stepwise backward multivariable regression analyses were done for adjustments. BMI, BMI≥25 kg/m2, WHR and abdominal obesity were set as outcome variables. Sleep variables, socioeconomic variables, lifestyle variables, health variables and biological variables were used as independent variables.

The associations between body composition variables and social jetlag and chronotype disappeared in the multivariate analyses when the other health variables, life-style variables and biological variables were introduced in the model. We found that the only sleep variable that was associated with BMI, waist circumference and WHR, was sleep duration. We also found that the optimal sleep length with respect to a low risk of obesity was 8–8.9 hours (see Table 4). Male short sleepers had a 70% higher risk of being in the BMI≥25 kg/m2 group (OR = 1.72, p = 0.010) and almost a doubled risk of having abdominal obesity (OR = 1.93, p = 0.014) compared to the 8–8.9 hours sleepers. Female short sleepers had a slightly lower risk of being in the BMI≥25 kg/m2 group compared to male short sleepers (OR = 1.64, p = 0.05), but sleep duration had no association with WHR or abdominal obesity for women.

**Table 4:** Overweight and abdominal obesity risk.

	OR^1^	SE	t/z	p	F/Chi2	R2
**MALE**						
**BMI≥25 kg/m2**	1.72	0.36	2.57	0.010	332.89	0.168
**Abdominal obesity** 2	1.93	0.52	2.47	0.014	289.83	0.136
**FEMALE**						
**BMI≥25 kg/m2**	1.64	0.29	2.83	0.005	370.41	0.158
**Abdominal obesity** 3	NS^4^					

The table shows risk of overweight or abdominal obesity between 8–8.9 h and <6 h sleepers.

1 Odds Ratio.

2 Waist circumference ≥102 cm.

3 Waist circumference ≥88 cm.

4 Not significant.

The model explained about 25% of the difference in BMI and 16% of the risk of having BMI≥25 kg/m2 for both genders.

Table 5 shows all the variables in the logistic regression analyses that were significant for being overweight (BMI≥25 kg/m2), with both genders analysed together.

**Table 5:** Factors related to overweight risk, both genders.

BMI≥25 kg/m2	Odds Ratio	SE	z	P	95% CI	
Sleep duration 8–8.9 h^1^	1.88	0.269	4.43	<0.001	1.42	2.49
Sleep duration 7–7.9 h^1^	1.19	0.104	2.02	0.043	1.01	1.42
Heart disease (no/yes)	2.10	0.779	2.01	0.045	1.02	4.35
Alcohol use ≥5 units/week	1.36	0.197	2.09	0.037	1.02	1.80
Triglycerides (mmol/l)	1.69	0.136	6.55	<0.001	1.45	1.98
Cholesterol (total) (mmol/l)	1.29	0.061	5.37	<0.001	1.18	1.42
Glucose (mmol/l)	1.17	0.065	2.78	0.005	1.05	1.30
Systolic blood pressure (mmHg)	1.01	0.003	3.63	<0.001	1.01	1.02
Diastolic blood pressure (mmHg)	1.01	0.006	2.32	0.020	1.00	1.03
Self-evaluated good health (no/yes)	0.74	0.080	−2.74	0.006	0.60	0.92
Sleeping pills weekly in the last month (no/yes)	0.57	0.147	−2.18	0.029	0.34	0.94
Education beyond high school (no/yes)	0.79	0.067	−2.79	0.005	0.67	0.93
Current smoker (no/yes)	0.50	0.055	−6.27	<0.001	0.40	0.62
HDL (mmol/l)	0.25	0.031	−11.03	<0.001	0.20	0.32
Exercise >2–3 times/week	0.73	0.063	−3.64	<0.001	0.62	0.87
Constant	0.11	0.056	−4.41	<0.001	0.04	0.30

All the variables in the logistic regression analyses that were significant for being in the BMI≥25 kg/m2 group.

1 Sleep duration compared to the <6 h sleep group (reference group).

## Discussion

Several studies have demonstrated an association between sleep duration and BMI, but without consensus with regard to the optimal sleep duration with respect to BMI and waist circumference. A methodological problem is represented by the fact that some studies have defined short sleep duration as ≤6 hours while others as ≤5 hours. Moreover, the reference sleep group has in some studies had a sleep duration between 7 and 8 hours [1], [10], [14], while others have used 7 to 9 hours sleep as the reference group [2].

In this latter part of The Tromsø Study, we found that even if 7–7.9 hour sleepers had the lowest WHR and the lowest prevalence of abdominal obesity, the optimal sleep length with regard to all body composition measures was actually between 8 and 9 hours, when all the life style factors, health factors, and biological factors were accounted for in the multivariate regression analyses. As shown in Table 5, exercise, education, self-evaluated good health, smoking and HDL-cholesterol level lowered the risk of being overweight. On the contrary, alcohol use, heart disease and total cholesterol and triglyceride levels were associated with increased overweight risk. More or less the same pattern was found for WHR and male abdominal obesity (results are not shown). Magee et al. [30] underlined the need to control for potentially confounding variables in this type of studies. In their study, Fogelholm et al. [37] included sex, age, mental health, smoking and education as confounding factors, while Kohatsu et al. [38] adjusted for sex, age, educational achievement, physical job demand, household income, depressive symptoms, marital status, alcohol consumption and snoring. In our study, we also included physical activity in leisure time, self-rated physical health, former and current heart disease and diabetes, blood pressure, use of medication, HSCL-10 score, coping, sleep variables and biological factors. One possible explanation for the difference in optimal sleep length in our study compared these other studies [37], [38] could be that we have included more relevant variables, which is a major strength of our study. Other strengths of the present study were the high number of participants and that height, weight, and measures of waist and hip were done by trained personnel, as self-reported height is often overestimated and weight underestimated [39], which could lead to underestimation of BMI.

More people with elevated body weight were found in the short sleeping group, and the risk of having BMI≥25 kg/m2 was about 80% higher in this group compared to moderate sleepers. Short sleeping also doubled the abdominal obesity risk for men, but not for women.

Roenneberg et al. found that beyond sleep duration, social jetlag was associated with increased BMI [18]. This was a large epidemiological study including more than 64,000 people aged 16–65 years, but the study did not include important predictors of body weight like educational level, health factors or diet. In another study, Baron et al. found that calories consumed after 8:00 PM may increase the risk of obesity, independent of sleep timing and duration [40]. In our study we did not find an association between elevated BMI and social jetlag or chronotype. In the present study we did not report data on diet, anyway some of the biological variables that were included (triglycerides, total cholesterol, HDL-cholesterol and glucose) could –at least in part- be taken to reflect participants' diet.

The associations between sleep duration and BMI are far from fully understood, and studies have looked for explanations other than short sleeping, sleepiness-related inactivity and excess energy intake. Short sleep (<5 h) has been associated with reduced levels of leptin (“anorexigenic hormone”) and elevated levels of ghrelin (“hunger hormone”), which could increase appetite and also BMI [41]. Spiegel et al. found the same leptin and ghrelin changes, together with alterations in glucose metabolism in persons sleeping <4 hours [42]. In our study, we excluded persons who reported sleeping <3 hours since BMI elevation always develops over time, and habitual sleeping <3 hours per night is likely to occur only over a short period of time. We have no data on appetite hormone levels, but the significantly higher BMI levels among short sleepers was found after controlling for confounding factors including (non-fasting) total-cholesterol, HDL-cholesterol, triglycerides and glucose levels.

In our study, associations between short sleep and elevated risk of overweight (BMI≥25 kg/m2) was found for both genders, and the association was slightly stronger for men. Studies have shown gender differences in sleep patterns, with women sleeping longer than men [43], as we have previously reported [28].

Stranges et al. found no association between short sleep duration and increased BMI or waist circumference in prospective analyses of data from the Whitehall II Study [14]. This is comparable to the findings from the CARDIA Sleep Study where there were no longitudinal associations between sleep measurements and BMI [44].

It has been pointed out that although there has been evidence for associations between obesity and short sleep duration, most short sleepers are unlikely to be obese, and most obese people are unlikely to be short sleepers [45]. In our study, as well as most of the studies referred to by Horne, the number of participants was high, sleep duration was self-reported by a questionnaire and BMI was measured. We found that people with normal weight slept significantly longer than those who were in the overweight group, but the difference was only about 5 minutes. Even if an inverse relationship between sleep duration and the risk of having an elevated BMI was found for both genders in our study, the difference in age corrected BMI was very small (1 BMI unit for men and 0.8 BMI units for women), similar to the difference in BMI between sleep groups referred by Horne. This difference in BMI is possibly not large enough to have a clinical significance.

The present study had some limitations that should be mentioned. The MCTQ is based on self-reported data, and the recordings for sleeping behavior were the participants' own estimates and probably not the exact times. It is important to keep this in mind when analyzing and comparing the sleep duration data. Cournot et al. found independent and positive associations between BMI and the number of naps per week in women [22]. Napping during the day was not reported in our study, but in a study by Ursin et al., 26.5% of the men and 28.9% of the women reported napping several times a week or more [46]. Women had slightly longer naps than men, 48 minutes in women versus 45 minutes in men. We have no reason to believe that this phenomenon was less common among the participants in the Tromsø study than in the Hordaland study. If we anticipate that the napping is more common in short sleepers, and that napping was more common and slightly longer in women, short sleep would be even more strongly associated with an increased BMI in women. Unfortunately, we do not know if this is the case in our study.

The Tromsø study had low response rates in the age group 30–34 and in subjects aged 70 years or more, but findings from a study on non-responders evidenced that selection according to sociodemographic variables had little impact on prevalence estimates for participants compared to non-participants [47]. This, together with our exclusion of participants over 65 years, suggests that the low response rate in the age group 30–34 years had a minor impact on the health variables in our study.

A possible hypothesis for the difference in optimal sleep duration in this study compared to other studies is that subarctic people have different sleeping patterns than other populations because of the extreme photic conditions in this area, and that this could have an impact on BMI. This ought to be investigated in further longitudinal studies in subarctic populations.

## Conclusions

In our subarctic population, the optimal sleep duration with regard to minimal risk of overweight and abdominal obesity was 8–9 hours, which is one hour longer compared to findings from other studies. Short sleepers (both genders) had about 80% increased risk of being overweight, and men had a doubled risk of having abdominal obesity. We found no associations between chronotype or social jetlag and BMI or abdominal obesity, when we took a range of life-style, health and biological variables into consideration.
